# Diastolic dysfunction and diastolic heart failure: diagnostic, prognostic and therapeutic aspects

**DOI:** 10.1186/1476-7120-3-9

**Published:** 2005-04-04

**Authors:** Maurizio Galderisi

**Affiliations:** 1 Division of Cardioangiology with CCU Department of Clinical and Experimental Medicine "Federico II" University, Medical School Napoli, Italy

**Keywords:** Diastolic dysfunction, Diastolic heart failure, Left ventricle, Cardiac catheterization, Doppler echocardiography

## Abstract

Left ventricular (LV) diastolic dysfunction (DD) and diastolic heart failure (HF), that is symptomatic DD, are due to alterations of myocardial diastolic properties. These alterations involve relaxation and/or filling and/or distensibility. Arterial hypertension associated to LV concentric remodelling is the main determinant of DD but several other cardiac diseases, including myocardial ischemia, and extra-cardiac pathologies involving the heart are other possible causes. In the majority of the studies, isolated diastolic HF has been made equal to HF with preserved systolic function (= normal ejection fraction) but the true definition of this condition needs a quantitative estimation of LV diastolic properties. According to the position of the European Society of Cardiology and subsequent research refinements the use of Doppler echocardiography (transmitral inflow and pulmonary venous flow) and the new ultrasound tools has to be encouraged for diagnosis of DD. In relation to uncertain definitions, both prevalence and prognosis of diastolic heart failure are very variable. Despite an apparent lower death rate in comparison with LV systolic HF, long-term follow-up (more than 5 years) show similar mortality between the two kinds of HF. Recent studies performed by Doppler diastolic indexes have identified the prognostic power of both transmitral E/A ratio < 1 (pattern of abnormal relaxation) and > 1.5 (restrictive patterns). The therapy of LV DD and HF is not well established but ACE-inhibitors, angiotensin inhibitors, aldosterone antagonists and β-blockers show potential beneficial effect on diastolic properties. Several trials, completed or ongoing, have been planned to treat DD and diastolic HF.

## Introduction

Heart failure (HF) is a clinical syndrome whose symptoms and signs are due to increased extravascular water and decreased tissue / organ perfusion. The definition of the mechanisms inducing HF needs the measurement of both left ventricular (LV) systolic and diastolic function since HF may occur in patients with either normal or abnormal LV ejection fraction (EF) [1].

Arterial hypertension is the most common risk factor for HF in the general population and myocardial infarction, LV hypertrophy (LVH) and valve heart disease represent predictors of subsequent HF in hypertensive patients of both genders [2]. The progression of hypertensive cardiomyopathy towards HF includes serial LV changes – LV concentric remodelling and LVH – whose prognostic role is recognized [3-5]. In presence of these LV geometric abnormalities, deep modifications of LV diastolic properties occur. These modification are globally defined as LV diastolic dysfunction (DD) and include alterations of both relaxation and filling [6,7] which can precede alterations of LV systolic function and be *per se *main determinants of symptoms and signs of HF. Several other cardiac pathologies as well as extra-cardiac diseases involving secondarily the left ventricle can also affect myocardial diastolic properties and determine LV DD.

LV DD and diastolic HF, that is the symptomatic DD, represent clinical entities which can be described at different levels, from the hystologic and ultrastructural features to the clinic manifestations and diagnostic instrumental findings, until the prognostic and therapeutic aspects. The growing interest for DD and for diastolic HF has been developed gradually in the last 10–15 years. It rises mainly from the advancement of non invasive imaging tools, above all Doppler echocardiography, which, to date, allows easy and repeatable identification of LV diastolic abnormalities, and by the growing impulse of pharmaceutical industry, at constant search of new therapeutic applications. In relation to the increase of the average life and the future projections which suggest HF as the most important pathology of the new millennium, particularly in the elderly population, it has to be understood how diagnosis, prognosis and therapeutic management of DD represent very attractive perspectives.

## Physiology of diastole

Although in normal hearts the transition from contraction to relaxation begins much more before LV end-systole, i.e., at 16% to 20% of the ejection period [8,9] and even prior to aortic valve opening when LV contractility is severely impaired (9), the traditional definition of diastole (in ancient Greek language the term διαστολε means "expansion"), includes the part of the cardiac cycle starting at the aortic valve closure – when LV pressure falls below aortic pressure – and finishing at the mitral valve closure. A normal LV diastolic function may be clinically defined as the capacity of the left ventricle to receive a LV filling volume able in its turn to guarantee an adequate stroke volume, operating at a low pressure regimen.

In merely descriptive terms, diastole can be divided in 4 phases [10]:

1. *Isovolumetric relaxation*, period occurring between the end of LV systolic ejection (= aortic valve closure) and the opening of the mitral valve, when LV pressure keeps going its rapid fall while LV volume remains constant. This period Is mainly attributed to the active LV relaxation, with a lower, variable contribution of elastic recoil of the contracted fibers;

2. *LV rapid filling*, which begins when LV pressure falls below left atrial pressure and the mitral valve opens. During this period the blood has an acceleration which achieves a maximal velocity, direct related to the magnitude of atrio-ventricular pressure, and stops when this gradient ends. This period represents a complex interaction between LV suction (= active relaxation) and visco-elastic properties of the myocardium (= compliance);

3. *diastasis*, when left atrial and LV pressures are almost equal and LV filling is essentially maintained by the flow coming from pulmonary veins – with left atrium representing a passive conduit – with an amount depending of LV pressure, function of LV "compliance".

4. *atrial systole*, which corresponds to left atrial contraction and ends at the mitral valve closure. This period is mainly influenced by LV compliance, but depends also by the pericardial resistance, by the atrial force and by the atrio-ventricular synchronicity (= ECG PR interval).

Cardiac catheterization allows to assess the pressure-volume relation along the overall cardiac cycle. Among the various hemodynamic measurements, τ (= time constant of the isovolumic-pressure decline) and DP/DV ratio, expression of LV end-diastolic myocardial stiffness, are the main invasive measurements of LV diastolic function [10]. On the other hand, Doppler recording of transmitral and pulmonary venous flow measure flow velocities and time intervals, whose variations occur in relation to analogous variations of left atrial and LV pressures [11,12]. Thus, Doppler parameters provide important information about dynamics of LV filling and LV diastolic properties during disease evolution or improvement [13].

## Ultrastructural features of diastolic dysfunction

The extracellular matrix (ECM), corresponding to fibrillar collagen, is an important structure for processes of both myocardial contraction and relaxation. It facilities the arrangement of the cardiomyocites into the most suitable allocation for the development of force and shortening, giving a substantial support to the maintenance of an effective myocardial performance [14]. The myocardial remodelling is accompanied by changes of myocardial cell factors but also of the ECM where fibroblast proliferation, alteration of the collagen network and increase in interstitial and perivascular collagen are strongly promoted by renin-angiotensin-aldosterone system [15]. ECM has, therefore, to be considered a dynamic entity playing a fundamental role into the myocardial adaptation to physiologic and pathologic stress [14]. ECM undergoes an intense turnover, due to balanced action of metalloproteases, proteolytic enzymes activated by several factors including also BNP, and tissue inhibitors counterbalancing the activity of metalloproteases [16]. Thus, if the collagen destruction alters both geometry and function of contractile myocardium throughout an up-regulation of metalloproteases, on the other hand myocardial fibrosis occurs because of an imbalance where collagen deposition prevails over its degradation. According to the ultrastructural view, we can hypothesize two opposite pathologic conditions: the first one, when the collagen loss, e.g, after acute myocardial infarction, deprives myocardium of its indispensable support structure, thus inducing a reduction of myocardial systolic function; the second one, when the accumulation of the same collagen, main component of myocardial fibrosis, determines both systolic and diastolic myocardial dysfunction. In this context not only the total amount of collagen is main determinant of LV diastolic stiffness but also distribution, configuration, disorganization of collagen fibers (cross-hatching), and ratio of collagen type I to collagen type III play an important role [14].

## Clinical, hemodynamic and diagnostic and aspects of diastolic dysfunction

In the clinical setting the coexistence of systolic and diastolic dysfunction in patients with symptomatic HF occurs very often. In fact, LV stiffness (or compliance) is related to the length of myocardial fibers, reflecting in its turn on LV end-diastolic dimensions. LV diastolic function, through the influence on left atrial and capillary wedge pressures, determines the onset of symptom in patients with prevalent LV systolic dysfunction too.

In parallel to the ultra-structural level, the clinical progression of HF may follow two different routes. In the first one, as it happens after acute myocardial infarction, post-infarction LV dilation (= remodelling) leads to ***systolic dysfunction and/or systolic heart failure***. In the second one, LV structural abnormalities (= LV concentric geometry) induce functional alterations of DD. When ***diastolic dysfunction ***becomes symptomatic – that is, when dyspnoea occurs – ***diastolic heart failure ***rises.

The majority of patients affected by isolated diastolic HF show symptoms not at rest but in relation to stress conditions (II NYHA class). Symptoms can be induced or worsened by, firstly, physical exercise but also by events as anaemia, fever, tachycardia and some systemic pathologies. In particular, tachycardia reduces the time needed for global LV filling, thus inducing an increase of left atrial pressure and consequent appearance of dyspnoea, because of accumulation of pulmonary extravascular water.

**Figure 1:**
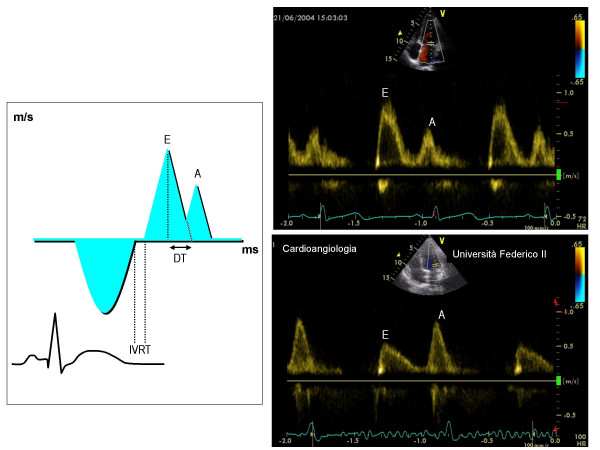
In the left screen, methodological outline for the measurement of Doppler transmitral indexes of diastolic function. In the right screen, normal diastolic pattern (upper part) and pattern of abnormal relaxation (lower part). A = atrial velocity (m/s), DT = deceleration time of E velocity (ms), E = early diastolic velocity (cm/s), IVRT = isovolumic relaxation time (ms)

**Figure 2:**
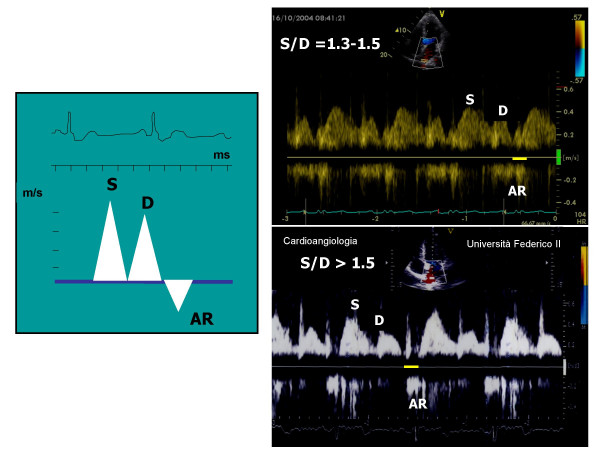
In the left screen, methodological outline for the measurement of pulmonary veins flow. In the right screen, normal pulmonary veins flow pattern (upper part) and pattern of abnormal relaxation (lower part).

**Figure 3:**
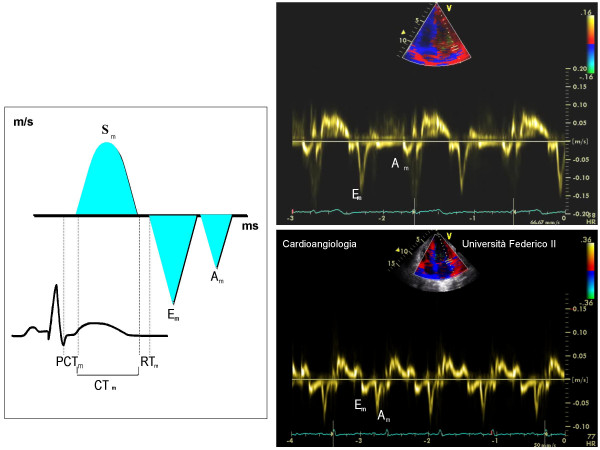
In the left screen, methodological outline for the measurement of Tissue Doppler indexes. In the right screen, normal myocardial diastolic pattern (upper part) and pattern of abnormal myocardial relaxation (lower part). A_m _= myocardial atrial velocity (cm/s), CT_m _= myocardial contraction time (ms), DT_m _= myocardial deceleration time of E_m_(ms), E_m _= myocardial early-diastolic velocity (cm/s), PCT_m _= myocardial pre-contraction time (ms), RT_m _= myocardial relaxation time (ms).

The diagnosis of HF can be performed obviously by the simple clinical examination but the identification of the diastolic origin needs an instrumental assessment. In fact, the objective examination of patients with diastolic HF allows to notice the same signs occurring for systolic HF and even the thoracic X-ray can not be useful to distinguish the two entities. ECG can show signs of LVH, due to hypertensive cardiomyopathy or other causes. DD may be asymptomatic and, therefore, identified occasionally during a Doppler echocardiographic examination (Figure 1). The diagnostic importance of this tool rises from the high feasibility of transmitral Doppler indexes of diastolic function, shown even in studies on population [17], such to be suitable and accurate also for serial evaluations over time. To date, standard Doppler indexes may be efficaciously supported by the evaluation of pulmonary venous flow [18] (Figure 2) and by new ultrasound technologies as Tissue Doppler [19] (Figure 3) and color M-mode derived flow propagation rate [20]. The application of maneuvers (Valsalva, leg lifting) [21,22] to Doppler transmitral pattern and/or different combination of standard transmitral Doppler with the new tools (ratio between atrial reverse velocity duration and transmitral A velocity duration, ratio between transmitral E peak velocity and Tissue Doppler derived Em of the mitral annulus or flow propagation velocity [Vp]) are sufficiently reliable to predict capillary wedge pressure and to distinguish accurately variations of LV end-diastolic pressure [23,24]. Some of these tools are effective even in particular situations as sinus tachycardia [25] and atrial fibrillation [26] while the pulmonary venous flow or the Valsalva maneuver applied to transmitral inflow has to be preferred in the case of mitral valve prosthesis and aortic valve regurgitation [27]. In addition, Tissue Doppler is also able to "read" the percentage of myocardial fibrosis [28], *primum movens *of DD. Alone or, better, combined together, these tools permits to recognize normal diastole as well as to diagnose and follow the progression of DD from the pattern of abnormal relaxation (grade I of DD) until pseudonormal (grade II) and restrictive (grade III-IV) patterns (Table 1).

**Table 1:** Doppler echocardiographic patterns of current echocardiographic tools in relation to the grading of LV diastolic dysfunction

**Parameter**	**Normal pattern**	**Pattern of abnormal relaxation (Grade I)**	**Pseudonormal pattern (Grade II)**	**Restrictive patterns (Grades III-IV)**
E/A	>1	<1	1 – 2	≥2
DT (ms)	160 – 210	>220	150 – 200	<150
IVRT (ms)	70 – 90	>95	60 – 95	<60
S/D	1.3 – 1.5	1.6 – 2.0	<1	0.40 – 0.60
AR (m/sec)	0.22 – 0.32	0.21 – 0.28	≥0.35	≥0.25
E_m _(cm/sec)	>8	<8	<8	<5
Vp (cm/sec)	>55	<45	<45	<35
E/E_m_	< 8			> 16
E/Vp				> 2.5

AR = Atrial retrograde velocity, DT = Deceleration time, E/A = Transmitral E/A ratio, E/E_m _= Transmitral early diastolic velocity to myocardial early diastolic velocity of lateral mitral annulus by Tissue Doppler, E_m _= myocardial early diastolic velocity by Tissue Doppler at lateral mitral annulus, IVRT = Isovolumic relaxation time, = Systolic velocity to diastolic velocity ratio by pulmonary veins assessment, Vp = Velocity propagation

**Figure 4:**
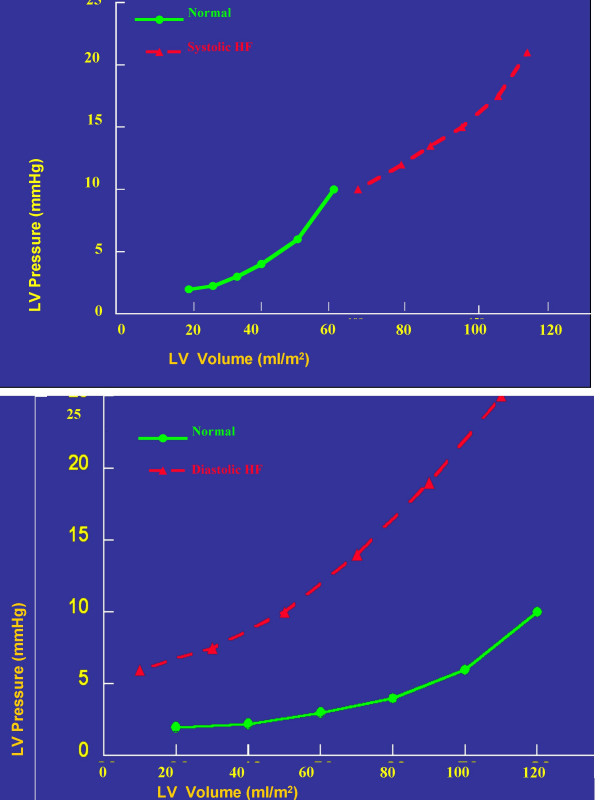
Pressure-volume loops in systolic HF (upper part) and diastolic HF (lower part). The continuous black line refers to normal, the interrupted red line to the pathologic condition.

By the hemodynamic point of view, the differences between diastolic and systolic HF are expressed by the pressure-volume loop (Figure 4) [29]. When systolic HF occurs, increased LV filling pressures correspond to increased LV volumes, with a displacement of the loop upon and at right. In the case of diastolic HF, the increase of LV filling pressures occur in the presence of normal or even reduced LV volumes, thus moving the loop up and to the left. It is obvious that in the more advanced stages of HF, diastolic and systolic dysfunction coexist.

## Determinants of diastolic dysfunction

LV DD develops in several cardiac diseases [30] as well as in extra-cardiac pathologies involving the heart (accumulation diseases as amyloidosis, thyroid disorders, acromegaly and others) [31,32] and in myocardial ischemia due to coronary artery stenosis or even to isolated dysfunction of coronary microcirculation [33]. However, the main cause of DD is arterial hypertension [5-7]. Overweight and obesity, often coexisting with the same hypertension, deeply affects LV diastolic function, forcing the left ventricle to a working overload [34]. In this view, DD represents one of the cardiac consequences of pluri-metabolic syndrome, where arterial hypertension, obesity, glucose intolerance and hypertrygliceridemia cohabit in the same subject, having their common matrix in the insulin resistance. High levels of insulin resistance, often evident in arterial hypertension [35], are positively associated with the prolongation of isovolumic relaxation time, independent of LV geometric changes and of increased afterload [36]. The alteration of diastolic isovolumic relaxation is probably due to an increment of intracellular calcium, which has been observed in insulin resistant hypertensives and is induced in its turn by an abnormal re-uptake of calcium by sarcoplasmic reticulum [37]. Also the hormones produced by adipose tissue, as leptin – involved into the control of body weight throughout food absorption and energy-giving cost – negatively affects LV diastolic function [38]. The association of arterial hypertension and diabetes mellitus worsens furher Doppler indexes of LV diastolic function as shown into the population of the Strong Heart Study [39].

It is controversial whether LV DD is necessarily accompanied on the development of LVH or rises up independent of it [5-7,40-43]. It is true that DD is a direct sequence of pressure overload, associated to elevated 24-hour blood pressure [40] and even more to the increment of night-time diastolic blood pressure [43]. Recent studies point out that the diastolic abnormalities of hypertensive patients are related to inappropriately high levels of LV mass, disproportionate to the hemodynamic load predicted by the individual body size and cardiac load, more than to the values of LV mass which traditionally define LVH [44]. Inappropriately high LV mass is a potent predictor of cardiovascular risk in hypertensive patients, in presence as in absence of clear cut LVH [45]. The concept of DD onset preceding the appearance of LVH is consistent with the observation that BNP, whose levels grow gradually with the progression of DD grading (from abnormal relaxation until restrictive Doppler patterns) [46], are increased in patients with diastolic HF independent of the magnitude of LV mass [47]. Even a new ultrasound technology as Tissue Doppler supports the hypothesis of an early evidence of DD in hypertensive heart: myocardial DD (= E_m_/A_m _ratio < 1 at the level of multiple LV walls in the apical views) is detectable before the appearance of the abnormalities involving LV transmitral inflow and is uniform in non hypertrophic patients while it becomes prominent at the septum in presence of overt LVH [48]. Table 2 reports the differential characteristics involving in the meantime the myocardial ultra-structure and LV geometry in systolic and diastolic HF: it is clear that diastolic HF is associated to both increase of collagen amount and LV concentric geometry [49]. This concept is further supported by the HyperGEN study where delayed LV relaxation is independently associated with concentric LV geometry in 1384 hypertensive participants including obese and diabetic patients [50].

**Table 2:** Differential characteristics of LV geometry between systolic and diastolic HF (modified from Zile MR [49]).

**Characteristic**	**Systolic HF**	**Diastolic HF**
LV volume	↑ ↑↑	N (o ↓)
LV mass	↑	↑
LV geometry	Eccentric	Concentric
Cardiomyocites	↑ Length	↑ Diameter
Extracellular Matrix	↓(o ↑ o N) Collagen	↑↑ Collagen

HF = heart failure, LV = left ventricular, N = normal

## Definition and classification criteria for diastolic HF

The evidence of acute HF in absence of overt LV systolic dysfunction rises by the experience of Gandhi and coworkers [51]: thirty-height hypertensive patients affected by pulmonary oedema, undergoing echocardiographic examination during the acute episode and after clinical stabilization respectively (1–3 day after), did not show significant variations of LV EF (50 ± 15% and 50 ± 13% respectively, NS) and of wall motion score index between the two examinations. This clinical condition, defined as **heart failure with preserved systolic function **or, better, with normal EF, has been made equal to **isolated diastolic heart failure**. A truly correct definition of this clinical entity should, however, be done on the grounds of direct estimation of LV diastolic function and establishment of reference normal values. Strong controversy has been developed in the previous years about this issue, with opposite scientific positions. The American point of view, corresponding to the Framingham Heart Study investigators, has sustained the concept that diastolic HF is "definite" only when an invasive hemodynamic assessment shows diastolic alterations in the temporal proximity of the acute episode [52]. On the other hand, the European point of view (European Group on Diastolic Heart Failure) has defined diastolic HF according to criteria including clinical examination, echocardiographic assessment (normal EF) and Doppler indexes (derived by both transmitral inflow e pulmonary veins flow), whose normal partition values are referred for age ranges [53] (Table 3). Despite the obvious superiority of the invasive technique [54], it has to be taken into account that the need of cardiac catheterization for establishing a definite diagnosis of diastolic HF raises practical and even ethical issues. Practical issues are related to the low priority such examination would have in a cath-lab overloaded by coronary procedures and to the poor interest of hemodynamists in the assessment of indexes of LV diastolic function. Ethical concern lies upon the fact that the present reliance on echo-Doppler examination of LV diastolic function makes cardiac catheterization an useless invasive procedure to this end, except very particular cases. Moreover, if it is true that the prevalence of abnormal Doppler indexes (from 38% of isovolumic relaxation time to 64% for deceleration time) is much lower to that showed by the more reliable invasive measurements (92 % for LV end-diastolic pressure and 79 % for τ) [55], is also true that this can be, at least partially, due to the confounding influence of physiologic variables as age [56] and heart rate [57]. In this view, reference normal values of Doppler indexes of LV diastolic function should be done considering ranges of both age and heart rate. It is now current opinion that the diagnosis of diastolic HF can be made even without measurement of diastolic function if three criteria are present: 1) symptoms and signs of HF (Framingham criteria), 2) LV EF> 50%, and 3) ability to rule out mitral stenosis, pericardial disease, and non cardiac causes of dyspnoea, oedema and fatigue [58]. Recent evidences further sustain the definite role of Doppler echocardiography to diagnose diastolic HF [59,60].

**Table 3:** Criteria for diastolic HF according to the European Society of Cardiology (53).

Signs and symptoms of HF	Effort dyspnoea, Hortopnoea, III-IV tones, Pulmonary rales
Normal or mildly reduced LV systolic function	EF ≥ 45 % e LVIDDi > 3.2 cm·m ^-2^
Evidence of abnormalities LV of relaxation/filling and/or distensibility	IVRT _<30 years _> 92 ms, _30–50 years _> 100 ms, _>50 years _> 105 msc E/A<1 + DT>220 ms + S/D<1.5 _<50 years_ E/A<0.5 + DT>280 msec + S/S>2.5 _>-50 years_

DT = deceleration time of E velocity, E/A = ratio of early diastolic velocity to atrial velocity, EF = ejection fraction, HF = heart failure, IVRT = isovolumic relaxation time, LV = left ventricular, LVIDDi = left ventricular internal diastolic diameter index, S/D = ratio of systolic to diastolic velocity of venous pulmonary veins

To date, however, no certain definition of diastolic HF exists and the recognition of its existence is not unanimously accepted [49]. Studies performed by both standard Doppler echocardiography [61] and Tissue Doppler [62,63] demonstrated how sub-clinic alterations of myocardial systolic function are already overt in diastolic HF. Because of the use of LV EF is a rather insensitive indicator of true LV myocardial contractility, the assessment of LV long-axis function by the simple M-mode of the mitral lateral annulus could help to identify initial LV systolic dysfunction [64]. Finally, it has also to be taken into account how concomitant variables, including obesity, chronic obstructive lung disease and even myocardial ischemia, can be confounding factors leading to "false" diagnosis of diastolic HF, particularly in the elderly population [65].

## Prevalence of diastolic HF

The studies performed until now have assessed above all the prevalence of HF with normal EF, using standard echocardiography without Doppler. In a first meta-analysis of 1995, the investigators of the Framingham Heart Study [66] showed wide variability in the prevalence of this kind of HF (range = 13–74%) while a subsequent study involving the Framingham offspring cohort pointed out a 51% prevalence of overall HF [67]. Very recently, Hogg et al collected ten "cross-sectional" studies on population, in the United States as in several European countries, and found very high variability of HF with normal EF. The explanation of this variability is related mostly to different age and gender of participants. It has to be considered that this kind of HF is particularly frequent in the elderly population, occurs more often in the female gender and is associated much more with arterial hypertension and atrial fibrillation than to coronary heart disease [68]. The data collected between 1995 and 1999 from Italian Network on Congestive Heart Failure (IN-CHF) are strongly consistent with these results [69]. The choice of different cut-off points for normal LV EF can be an additional reason of variability for the prevalence of diastolic HF in the above mentioned studies.

## Prognosis of diastolic heart failure

Great heterogeneity exists also for results in prognosis of diastolic HF. By the Framingham meta-analysis the annual mortality varies from 1.3% to 17.5% [66]. This wide variability depends by several factors including first of all, the modality used to classify this kind of HF – mostly according to the evidence of normal EF – but also age and follow-up duration. In a study by registry on 1291 hospitalized patients (70) the mortality was lower in patients with EF ≥ 50% than in those with EF ≤ 39% (OR = 0.69 95% CI 0.49–0.98, p = 0.04) . The Framingham offspring cohort informed that the rate of death after 5 years is 68% in patients with HF and normal EF in comparison with 82% of systolic HF, with a mortality, however, four times greater than that presented by healthy subjects [67]. Although Senni et al (71) did not find difference of mortality between the two kinds of HF in a 4-year follow-up of a population with mean age of 78 years, the analysis of Hogg and coworkers, assembling the results of recent cohort studies performed on patients hospitalized for HF, noticed how the percentage of mortality for patients with HF and normal EF, mild during the first year and half, becomes similar to that of systolic HF after 5–6 years of follow-up (68). It is worthy of note the recent study of Badano and coworkers who, using the ESC criteria to identify diastolic HF in 179 patients hospitalized with HF, do not observe significant difference in 6-month mortality in comparison with patients having prevalent LV systolic dysfunction [72].

**Figure 5:**
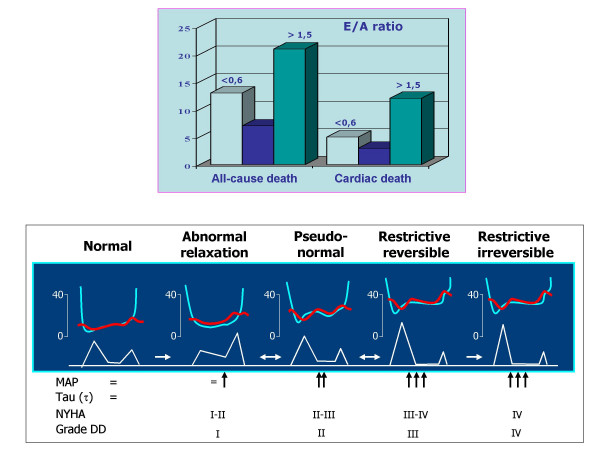
Results of overall and cardiac mortality in relation to transmitral E/A ratio in the Strong Heart Study [74] (upper panel)) and classification of DD grades (I-IV) according to Mayo Clinic suggestions [21] (lower panel). It can be observed a parallel behaviour between clinic progression and prognostic value of different grade of DD: the increment of mortality in Strong Heart Study has an "U" behavior, where E/A ratio <0.6 (grade I of DD) and >1.5 (grades II, III, IV) are both main predictors of mortality DD = diastolic dysfunction, NYHA = New York Heart Association, MAP = mean atrial pressure

Two important studied have finally pointed out the prognostic value of Doppler indexes of LV diastolic function and in particular of transmitral E/A ratio [73,74]. The first one, the PIUMA study [73], evidenced that the pattern of abnormal relaxation (= E/A ratio lower than that predicted individually by age and heart rate) increases the risk of cardiovascular events (odds ratio 1.57, 95% CI 1.1-2,18, p < 0.01) in a population of 1839 hypertensive patients during a 11 years follow-up. This prognostic value is independent of the effect of LV mass and even of ambulatory 24-hour blood pressure. In the second one, the Strong Heart Study [74], by a 3-year follow-up on a population of 3008 American Indians, a transmitral E/A ratio < 0.6 (= likewise pattern of abnormal relaxation) is associated to a doubled increase of mortality risk – despite not independent of other covariates – and an E/A ratio > 1.5 (= likewise pattern pseudonormal / restrictive) is associated to an threefold increase of cardiac mortality, which is also independent of several confounders including LVH. This result is consistent with the findings of the Framingham Heart Study, where an "U" relation between transmitral A velocity and risk of atrial fibrillation is detectable, and the arrhythmia appears independently associated with both A velocity increase (= abnormal relaxation) and E/A ratio increase (pattern pseudonormal / restrictive) [75]. These two studies, in particular the Strong Heart Study by data on mortality, are very consistent with the physiopathologic point of view of the Mayo Clinic investigators, who created an ingenious classification of Doppler-.derived DD some years ago [21]. In this classification, the pattern of abnormal relaxation (grade I of DD) and both reversible and non reversible restrictive patterns (grade III and IV respectively) are at opposite sides in the clinical progression towards the end stages of HF while the pseudo-normal pattern has an intermediate, but clinically crucial, position (Figure 5). In view of these findings and combining the value of the prognostic studies, we can suppose that the relatively long time (5–6 years) needed to assimilate the prognosis of diastolic HF to that of systolic HF depends mainly by the transition from the initial grade of DD, when the pattern of abnormal relaxation prevails and dyspnoea is overt only during exercise, to the more advanced stages, when the high LV end-diastolic pressure is associated to "end-stage" HF.

## Therapy of DD and diastolic HF

The objectives of the therapy for LV DD include the improvement of hemodynamic conditions, concerning both preload and afterload. The volume overload, such to induce episodes of acute HF, can be prevented or reduced by hypo-saline diet or also by a moderate diuretic administration.

Conceptually, both ACE-inhibitors and angiotensin-inhibitors can exert a beneficial effect on DD, since they reduce both afterload and preload, induce regression of LVH and decrease of myocardial interstitial fibrosis [70]. Also the aldosterone antagonists, as sprironalattone [76] and canrenone [77], able to reduce the myocardial fibrosis, can be suitable to this aim.

When DD is overt, it is also important to control heart rate and avoid tachycardia. β-blockers, and, with a lower extent, calcium antagonist verapamile, can be particularly useful. Lower heart rate induces prolongation of LV filling time, allowing to counterbalance the resistance to the diastolic inflow of a stiffened left ventricle. Last generation β-blockers (carvedilol, nebivolol), provided of vasodilation activity, could be particularly indicated for the management of DD. A recent study has tested the ability of nebivolol on 26 patients affected by HF and normal EF, in comparison with the traditional atenolol, combining both invasive hemodynamic and Doppler echocardiographic assessment [78]. After six-month therapy, nebivolol much more than atenolol induced increase of both E/A ratio (from 0.79 ± 0.13 a 0.91 ± 0.11) from 0.84 ± 0.12 a 0.89 ± 0.15) (p < 0.004) and cardiac index and reduction of "wedge" pressures, both at rest and during exercise.

On these grounds, pharmaceutical industry has planned clinical trias to evaluate the prognostic impact of several drugs on diastolic HF. Indeed, the trials completed to date have been disappointing. The CHARM-2 (= Candesartan in Heart Failure – Assessment of Reduction in Mortality) [79] did not evidenced significant improvement of all-cause mortality, of cardiovascular mortality and of hospitalization rate for HF in the sub-set of patients with preserved systolic function, but the follow-up (37.7 months) was probably too short to verify the effects. Into SWEDIC (= Swedish Doppler-Echocardiographic study) [80], carvedilol, inducing a positive influence on transmitral E/A ratio in patients with heart rate > 71 bpm but not in those with HR < 71 bpm, did not exert any effects on the events. Among ongoing trials, the analysis of SENIORS (= Study of the Effects of Nebivolol Intervention on Outcomes and Rehospitalisation in Seniors with heart failure) [81] has not yet performed in the sub-set of patients with normal EF. PEP-CHF (perindopril versus placebo), I-Preserve (Irbesartan versus placebo) study and Hong Kong (rampiril, irbesartan, placebo) study have not completed to date [82].

New therapeutic fields for HF will be opened in view of the associations observed between the state of coronary microcirculation and LV diastolic function [33]. The beneficial effect of ACE-inhibitors on coronary flow reserve, reliable marker of coronary microcirculation function when stenosis of epicardial coronary arteries are not detectable , has been documented in relation to both blood pressure fall and reduction of LV mass. It has recently shown an improvement of transthoracic Doppler-derived coronary flow reserve after only 4-week anti-hypertensive nebivolol therapy, in relation to its endothelium-mediated vasodilation activity [85]. It has to hypothesize that the a restored function of the coronary microcirculation could be useful even for the improvement of DD in hypertensive heart [83,84].

## Conclusive implications

DD and diastolic HF are common entities in the clinical practice, particularly in hypertensive population. The diagnosis of diastolic HF can be considered in the presence of the signs of HF and normal EF (50% or more) but it should be usually supported by a Doppler examination. Diastolic HF is associated to four-fold increase mortality. If it is true that the mortality expectation is lower than in patients with systolic HF, it is even true that this difference has a trend to be blunted during long-term follow-up, with possible overlapping after 5.5 years or more. The therapeutic management of diastolic HF is, at least partially, empirical and several studies, ongoing or completed, have been planned to test the effects of ACE-inhibitors, angiotensin-inhibitors and β-blockers. The prevention of diastolic HF may be obtained by a better control of blood pressure values and of concomitant risk factors in hypertensive patients.

## Competing interests

### Financial competing interests

None.

### Non-financial competing interests

None.
